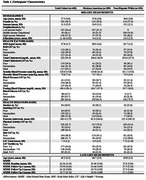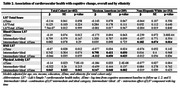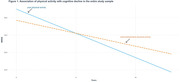# The impact of mid‐life cardiovascular health on cognitive change in a bi‐ethnic cohort

**DOI:** 10.1002/alz.092531

**Published:** 2025-01-09

**Authors:** Janette Vazquez, Rebecca Bernal, Hector A Trevino, Angel G Velarde, Monica Goss, Debora Melo van Lent, Helen Hazuda, Chen‐Pin Wang, Claudia L Satizabal

**Affiliations:** ^1^ Glenn Biggs Institute for Alzheimer’s & Neurodegenerative Diseases, University of Texas Health San Antonio, San Antonio, TX USA; ^2^ Medicine‐ Renal Diseases, University of Texas Health Science Center, San Antonio, TX USA; ^3^ Glenn Biggs Institute for Alzheimer’s & Neurodegenerative Diseases, San Antonio, TX USA; ^4^ Glenn Biggs Institute for Alzheimer’s & Neurodegenerative Diseases, University of Texas Health Science Center at San Antonio, San Antonio, TX USA

## Abstract

**Background:**

Vascular risk factors are known contributors to cognitive decline and dementia; however, their impact on cognitive change in diverse samples is understudied. In this study, we investigated the effect of the AHA Life’s Simple 7 (LS7) on cognitive decline in the bi‐ethnic San Antonio Longitudinal Study of Aging (SALSA) cohort.

**Method:**

We included 403 participants (mean age 57.9±3.8, 58.5% female, 52% Mexican Americans (MA), Table 1) from SALSA who were part of the San Antonio Heart Study (SAHS) launched in 1979. LS7 scores (score range 1‐13) integrated mid‐life data on diet, physical activity, body mass index (BMI), smoking, total cholesterol, blood pressure, and fasting blood glucose. We combined intermediate and ideal LS7 categories, using poor LS7 as the reference. The Mini Mental State Examination (MMSE) assessed global cognition at SALSA baseline (1992‐1996) and three follow‐up examinations. The study included individuals with complete data for computing LS7 scores, a baseline MMSE score, and at least one follow‐up MMSE exam. We used generalized estimating equations to explore the relationship between total LS7 score and single LS7 components with cognitive decline, both in the total sample and by ethnicity. The statistical model was adjusted for age, sex, education, income, and ethnicity (total sample only).

**Result:**

No relationships were observed between LS7 total score and cognitive decline. In the overall sample and among MA, meeting intermediate or ideal physical activity goals demonstrated a mitigating effect on cognitive decline (β±SE, 0.065±0.032, p = 0.041; 0.147±0.048, p = 0.002, respectively, see Table 2 and Figure 1). Additionally, maintaining blood glucose below 125 mg/dL was associated with slower cognitive decline (0.182±0.074, p = 0.014, Table 2) among non‐Hispanic White (NHW) but not among MA (0.035±0.098, p = 0.725, Table 2). In contrast, BMI under 30 was associated with accelerated cognitive decline (‐0.012±0.050, p = 0.025, Table 2) among MA but not among NHW (‐0.012±0.056, p = 0.836, Table 2).

**Conclusion:**

While the LS7 total score showed no significant association with cognitive decline in our bi‐ethnic cohort, notable associations were found for physical activity and blood glucose, highlighting their potential as effective preventative measures. Nonetheless, future multi‐ethnic studies are encouraged to replicate our findings, especially in larger sample sizes.